# Supplementary material for: Content-rich biological network constructed by mining PubMed abstracts
Source: BMC Bioinformatics. 2004 Oct 8;5:147. doi: 10.1186/1471-2105-5-147 (PMC528731; doi:10.1186/1471-2105-5-147)
Supplement: Additional File 2 — The original results of the above study (non-essential files are deleted to keep the file size under the limit set by BMC bioinformatics). [file 1471-2105-5-147-S2.bz2 › chilibotAdditionalFile2/dip05/644ID8642594E11031/html/F_G.html]

 


 **F** and **G** 
  
Found 14344 abstracts in PubMed, retrieved 05.  
 

 What does Google say? 
 PDF only 
| .edu only 

---

**Interactive relationship** (e.g. stimulation, inhibition, etc)

**Neutral relationship**- SpoIIAB, an anti sigma factor involved in regulation of sigma  **F** , is also shown to form a complex with sigma  **G**  in vitro.  Ref: 12867473 J Bacteriol, 2003

**Non-interactive relationship** (e.g. studied together, co-existance, homology, etc.)

- The ability of bPIV3 to function as a virus vaccine vector was explored further by introducing the RSV attachment  **G**  and fusion  **F**  genes into the bPIV3 RNA genome.  Ref: 12867647 J Gen Virol, 2003
- This recombinant virus expressed the RSV  **G**  and  **F**  proteins sufficiently to evoke a protective immune response in hamsters upon challenge with RSV or human PIV3 and to elicit RSV neutralizing and PIV3 haemagglutinin inhibition serum antibodies.  Ref: 12867647 J Gen Virol, 2003
- Some optimal physicochemical characteristics e. **g** . solvent exposed area, solubility of the molecule may be responsible for this special feature of  **f**  MLP at such a low level of phylogeny.  Ref: 12867161 Cell Biol Int, 2003
